# Supplementary material for: Integrated regulation of PKA by fast and slow neurotransmission in the nucleus accumbens controls plasticity and stress responses
Source: J Biol Chem. 2022 Jul 11;298(8):102245. doi: 10.1016/j.jbc.2022.102245 (PMC9386499; doi:10.1016/j.jbc.2022.102245)
Supplement: Supplemental Information [file mmc1.docx]

**Supplemental Information**

**Integrated Regulation of PKA by Fast and Slow Neurotransmission in Nucleus Accumbens Controls Plasticity and Stress Responses**

Rachel Thomas, Adan Hernandez, David R. Benavides, Wei Li, Chunfeng Tan, Alan Umfress, Florian Plattner, Ayanabha Chakraborti, Lucas Pozzo-Miller, Susan S. Taylor, and James A. Bibb

*Corresponding author. Email: [jbibb@uab.edu](mailto:jbibb@uab.edu)

**SUPPLEMENTAL FIGURES**


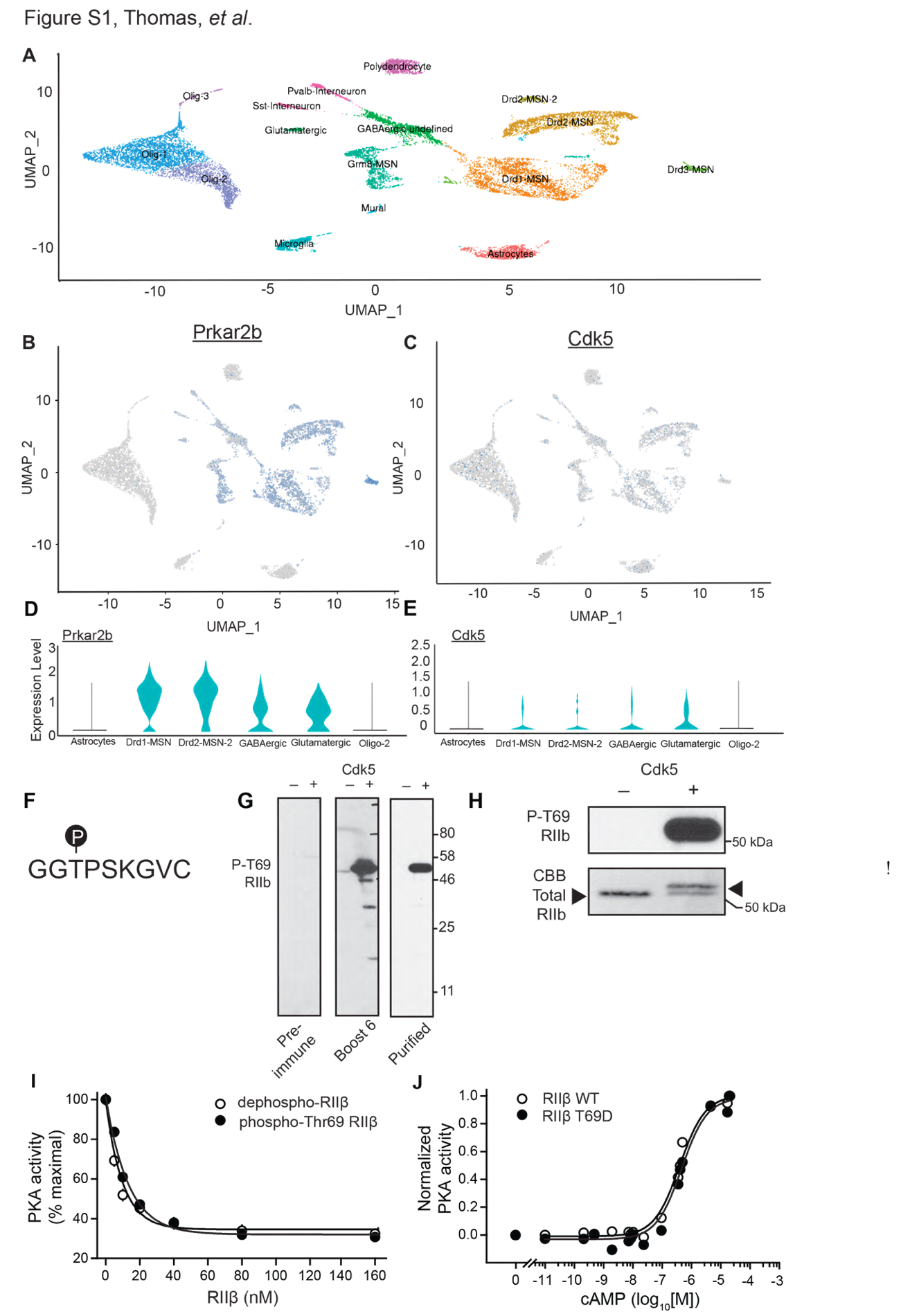


**Figure S1. Verification of phosphorylation-state specificity of phospho-T69 RIIβ antibody and assessment of the effect of phopsho-Thr69 on RIIβ/PKAcat or RIIβ/cAMP interactions.**

(A) Uniform Manifold Approximation (UMAP) displaying global cell-clustering across male Sprague-Dawley rats identifies major cell classes of the rat NAc (22) (B, C) Enrichment of *Prkar2b* and *Cdk5* within identified cell types is shown. (D, E) Violin plots indicating the average expression of marker genes in 6 identified cell types. (F) Amino acid sequence of the phospho-peptide used to generate the polyclonal anti-phospho-T69 RIIβ antibody. (G) Immunoblots of unphosphorylated (-) and *in vitro* phosphorylated (+) recombinant RIIβ with serum samples (preimmune, left; post-antigen boost 6, middle) and affinity purified (right) phospho-T69 RIIβ antibody. (H) Immunoblot (top) of unphosphorylated (-) and *in vitro* phosphorylated (+) recombinant RIIβ using phospho-T69 RIIβ antibody compared to total pure Coomassie Blue stained protein (bottom). Phosphorylation of RIIβ caused an upward shift in SDS-PAGE mobility. (I) Plot of PKA inhibition by dephospho- vs. phospho-T69 RIIβ. (J) Analysis of cAMP-dependent activation of PKA complexed with WT vs. T69D RIIβ. Data represent means ± S.E.M., n=4.

**Figure S2. Regulation of phospho-Thr69 RIIβ by NMDA is mediated through protein phosphatases PP2A, 2B, or 1.** (A) Quantitative immunoblot analysis of lysates from striatal slices treated with NMDA (25 μM, 5 min), in the absence or presence of the indicated protein phosphatase inhibitors cyclosporin A (CycloA, 10 μM, 1 h) or okadaic acid (OA, 1 μM, 1 h). Dotted lines denote where lanes were removed for exemplary blot comparisons. Data analyzed by one-way ANOVA (F(3,16) = 7.69 ^##^p = 0.002) followed by Tukey’s *post hoc*; **p* < 0.017 control vs. NMDA, n = 4-6*.* (B) Effects of protein phosphatase inhibition on basal T69 RIIβ phosphorylation state (CycloA, 10 μM, 1 h: **p* < 0.05, Student’s unpaired *t*-test, n=4-5) and (OA, 200 nM and 1 μM, 1 h). Dotted lines denote where lanes were removed for exemplary blot comparisons. Data analyzed by one-way ANOVA (F(2,10) = 32.93 ^##^p<0.0001) followed by Newman-Keuls *post hoc*, **p* < 0.05, control vs. 200 nM OA and ***p* < 0.01, control vs. 1 μM OA respectively, n = 4-5). All data are normalized means ± S.D.

**Figure S3. Inhibition of Thr69 RIIβ phosphorylation by siP *in vitro* and in intact brain.**

(A) Inhibition curve for phosphorylation of RIIβ by Cdk5 *in vitro*. Data represent means ± S.E.M.

(B) Immunoblot of analysis NAc slice lysates for effect of RIIβ siP treatment (10 µM, 1 h) in the presence of dopamine (10 µM, 15 min) on phospho- (P-S/T) Thr69 RIIβ, Ser845 GluA1, and Thr34 DARPP-32. Dotted lines denote where lanes were removed for exemplary blot comparisons. Data represent normalized means ± S.D., **p*<0.05, unpaired *t*-test, n=4.

**Figure S4. Positioning of electrodes for field recordings, I/O curves, and PPR.**

(A) Depiction of electrode placement for ventral striatal plasticity studies. (B) Effects of the D1 agonist SKF81297 (2 µM) on NAc LTP. Plot of fEPSP amplitudes with sample traces from a vs. b are shown (left) with summary plot (right), ***p* < 0.01, ****p* < 0.001, basal vs. LTP; *#p* < 0.05 control vs. SKF, unpaired *t*-test, n = 7. (C) RIIβ siP effects on HFS-induced LTP (left) in the presence of D1 agonist, SKF81297 with summary plot (right), **p*<0.05, basal vs. SKF; ***p* < 0.01, basal vs. RIIβ siP LTP, unpaired *t*-test, n = 8. (D) I/O curves and PPR for slices undergoing siP/SKF81297 protocol. These data correlate with data shown in panel C. Summary data represent means ± S.D.

**Figure S5. Stress response behavior in animals following peptide infusion.**

(A) Analysis of latency to immobility in the FST (one-way ANOVA (F(3,27) = 1.58 , p = 0.21)

(B) locomotor effects of bilateral intra-accumbens peptide infusion of control (scrambled), RIIβ siP, and/or PKI. Motor activity was recorded over the first 30 min following infusion (one-way ANOVA (F(3,27) = 0.71, p = 0.54) (C) Immobility time binned (2-min), comparing animals subjected to bilateral intra-accumbens infusion with scramble control peptide, siP (1 µl,100 µM), or ketamine (2 µg in 1 µl). Data represent means ± S.D., n = 6-8. Bins 4 to 6, and 6 to 8 were significant, two-way ANOVA (F (2,36) = 18.09 for time, p < 0.0001; F (2,18) = 8.52, for treatment, p = 0.0025; F (4, 36) = 8.21, for time and treatment interaction, ^####^p < 0.0001, followed by Bonferroni’s *post hoc*, ****p* <0 .0001, scramble peptide vs. RIIβ siP, *p =* 0.0002, scramble vs. ketamine respectively for bin 4 to 6, ***p* < 0.001 scramble vs. RIIβ siP and scramble vs. ketamine respectively for bin 6 to 8.
